# Supplementary material for: Comparing transcriptome profiles of human embryo cultured in closed and standard incubators
Source: PeerJ. 2020 Aug 11;8:e9738. doi: 10.7717/peerj.9738 (PMC7427541; doi:10.7717/peerj.9738)
Supplement: Supplemental Information 1 [file peerj-08-9738-s001.docx]

| Gene symbol | Length（bp） | Primers（5’→ 3’^，^） |
| --- | --- | --- |
| ELAVL1 | 168 | F:CCCTCTGGATGGTGGTGAAC |
|  |  | R:AAGCGGTTGAGAAAACGCAC |
| SMARCB1 | 169 | F:ACCTAACACTAAGGATCACGGA |
|  |  | R:GCTGTTCCTCTTGGCCTTCT |
| TPRAP | 123 | F:CGACATGAAGACGGTGGTGA |
|  |  | R:GACCAGGTCGGTTTACCCTG |
| RBM14 | 127 | F:CTGCGGCGACAAAATGAAGA |
|  |  | R:GGCGAACTGTTTCATGACGG |
| SLC5A12 | 191 | F:TGCCAGCAAATCTCAAACTGG |
|  |  | R:GAGCCCCAGAGATCAAACCC |
| ANGPT1 | 108 | F:TCCAGGAGCTGGAAAAGCAA |
|  |  | R:TGCAAAGATTGACAAGGTTGTGG |
| PRDM5 | 119 | F:GAGGACGCACACTGGAGAAA |
|  |  | R:ATTCTGCCAGGGGACGATTG |

**Supplementary Table S2. The primer list used in qPCR.**
